# Bronchoalveolar lavage for supporting acute cellular rejection in lung transplant recipients: A retrospective analysis

**DOI:** 10.1016/j.jhlto.2026.100582

**Published:** 2026-04-23

**Authors:** Carolin Steinack, Anna Hillinger, Jan H. Rüschoff, René Hage, Daniela Lenggenhager, Maurice Roeder, Silvan Vesenbeckh, Silvia Ulrich, Malcolm Kohler, Macé M. Schuurmans, Martina Haberecker, Thomas Gaisl

**Affiliations:** a Department of Pulmonology, University Hospital Zurich, Zurich, Switzerland; b Medical Faculty, University of Zurich, Zurich, Switzerland; c Department of Pathology and Molecular Pathology, University Hospital Zurich, Zurich, Switzerland

**Keywords:** Bronchoalveolar Lavage, Acute cellular rejection, Lung transplantation

## Abstract

**Background:**

Early detection of acute cellular rejection (ACR) is vital for improving long-term outcomes in lung transplant recipients (LTRs). Transbronchial biopsies are the gold standard for diagnosing ACR, but bronchoalveolar lavage (BAL) offers a less invasive alternative for early detection. This study examines the cytological and microbiological features of BAL, its correlation with ACR diagnosed via transbronchial cryobiopsy, and compares the effectiveness of pathology and hematology methods in analyzing BAL samples. Additionally, it assesses the value of mucosal biopsies for ACR detection.

**Methods:**

Transbronchial biopsies and corresponding BALs from January 2020 to March 2024 were analyzed retrospectively. ACR was diagnosed using cryobiopsy according to ISHLT histopathological criteria. Associations between BAL parameters and ACR were assessed using mixed-effects analysis.

**Results:**

Among 240 procedures from 143 LTR, 17.9% were diagnosed with ACR. Microbiological findings and mucosal biopsy analyses were not associated with ACR. In contrast, the relative lymphocyte counts in BAL (median 3% [IQR 2–5%]) was significantly associated with ACR (OR 1.07 [95% CI 1.02–1.12], p=0.004). A significant correlation between lymphocyte proportion and ACR severity was observed (r=0.385, p<0.001). A threshold of 5.7% lymphocytes achieved an ROC AUC of 0.715 (95% CI 0.613–0.817), with a positive predictive value of 66.7% and a negative predictive value of 76.5%.

**Conclusion:**

BAL lymphocytosis could support the diagnostic process of ACR. While mucosal biopsies and microbiological testing did not aid in ACR detection, BAL cytology provided relevant accuracy, particularly when assessed microscopically. These findings support incorporating BAL lymphocyte analysis into routine surveillance strategies for early ACR.

## Background

For patients with end-stage lung diseases, lung transplantation (LT) is often the only option for definitive treatment.^1^ Acute cellular rejection (ACR) is a common complication, occurring in 26.6% of patients within the first year following LT.^1^ Early detection of ACR remains difficult but crucial for ensuring appropriate treatment. Appropriate lung tissue obtained by transbronchial forceps or cryobiopsy is used to detect ACR.^2^, ^3^, ^4^ Nevertheless, transbronchial biopsies, as an invasive diagnostic method, continue to carry a risk of complications, including pulmonary hemorrhage and pneumothorax.^5^, ^6^ Bronchoalveolar lavage (BAL) is a standard diagnostic tool for identifying colonization or respiratory infection, and variation in BAL cell composition, and may suggest the presence of ACR.^7^ Examination of the cellular composition of the BAL fluid and the correlation with acute and chronic rejection has been an intense area of research interest.^8^, ^9^ Lymphocytosis greater than 20%, neutrophilia ≥12% without microbiological evidence of infection in the BAL, and the presence of eosinophils and basophils may indicate ACR.^10^, ^11^ Distinctive BAL cytokines, including IL-6, IL-8, IFN-γ, and TNF-α, were not linked with ACR.^12^ Current research is exploring novel biomarkers in BAL fluid to enhance the precision of ACR diagnosis.^13^, ^14^ It is unclear whether microscopic cytology or automated BAL analysis should be used after LT, and the role of mucosal biopsies (as a less invasive biopsy method) is unclear.^15^ Advanced techniques, including flow cytometry, are being developed to enable automated analysis of leukocyte subsets in BAL.^16^ Despite ongoing research into BAL fluid analysis, variable BAL fluid collection techniques and interpretations of the resulting data vary globally.^17^, ^18^ The role of large airway lymphocytic bronchitis obtained with endobronchial mucosal biopsy in the prognosis after lung transplantation is still under investigation.^19^, ^20^

This study aims to investigate the cytological and microbiological characteristics of BAL fluid comprehensively and examines their association with ACR, as diagnosed by transbronchial cryobiopsy. In addition, it compares the diagnostic performance of manual microscopic and automated analysis of BAL evaluation to determine their relative efficacy in detecting ACR. Furthermore, the potential utility of mucosal biopsies as an adjunctive or alternative approach for the detection and assessment of ACR in lung transplant recipients was investigated.

## Methods

### Patient selection

Routine surveillance bronchoscopies were scheduled at 1, 2, 4, 6, and 12 months post-LT and subsequently performed as needed based on clinical indications to assess patients for ACR. Additional bronchoscopies, referred to as clinically indicated procedures, were conducted in symptomatic lung transplant recipients (LTR), those exhibiting abnormalities on chest CT scans, or declining pulmonary function. All procedures were performed at the University Hospital Zurich, Switzerland between January 2020 and March 2024 and were retrospectively analyzed for this study. Patients were excluded from the study if they were under 18 years of age, had not provided consent for the procedure, or were on dual antiplatelet or anticoagulation therapy that could not be paused before the intervention. Relevant clinical, demographic, and pathological data, including findings from spirometry and bronchoscopy reports, were retrieved from electronic patient records. Additionally, BAL samples were analyzed using both manual and automated cell counting methods.

### Bronchoscopy and periinterventional care

All bronchoscopies were conducted in an inpatient setting. All LTR underwent BAL followed by three to four mucosal biopsies obtained with forceps biopsy in the second- to fourth-generation carina and two transbronchial cryobiopsies as recently described.^2^, ^4^ Once the bronchoscope is in a wedge position, three to four 50 ml aliquots of sterile isotonic saline were instilled in the middle lobe or Lingula and then manually suctioned for BAL through the flexible bronchoscope before mucosal or transbronchial biopsies were performed. The final BAL aliquot, at least 10 ml each, was utilized for manual and automated cell analyses to determine absolute and differential cell counts. BAL samples were sent in sterile containers within 1 h at room temperature to the clinical laboratory for cytological, hematological, microbiological including bacterial, fungal and mycobacterial cultures, as well as PCR for respiratory viruses (influenza, adenovirus, metapneumovirus, RSV, parainfluenzavirus, rhinovirus). PJP testing, galactomannan, Nocardia or CMV analysis were only requested when chest CT revealed any abnormalities suspicious for those infections. At least 10 ml of BAL were sent to microbiology laboratory. All biopsy techniques were performed during the same session, adhering to official guidelines.^5^, ^6^, ^21^, ^22^, ^23^ All procedures were conducted by respiratory physicians with at least 50 cryobiopsy procedures per year.

### Manual cell count of BAL

BAL samples were processed at the Department of Pathology and Molecular Pathology at the University Hospital Zurich, following standardized procedures. After volume measurement, samples were centrifuged for 10 min at 2500 rpm and the sediment was resuspended in 0.5 ml of 0.9% Saline. Total cell counts were determined using a Neubauer counting chamber with Tük’s solution. For cell differential counts, Papanicolaou (PAP) and DIFF-Quik stains were utilized. Diff-Quik stains were used to evaluate 2 x 100 cells using a microscope with a 40x objective, while PAP-stained slides were screened using standard cytological evaluation. Differential counts included macrophages, lymphocytes, neutrophils, eosinophils, mast cells and plasma cells. Additional special stains were applied when clinically indicated: Grocott stain for fungi and opportunistic pathogens, Ziehl-Neelson stain for acid fast bacilli (i.e. mycobacterium spp.), iron stain for siderosis evaluation and Sudan stain for the detection of lipid-laden macrophages.

### Automated cell count of BAL

BAL samples were analyzed in the Department of Hematology at the University Hospital Zurich, using native material. An automated cell count was performed with the Sysmex XN-series analyzer in the Body-Fluid-channel. Differential counts were assessed microscopically after Giemsa staining. Where clinically indicated, immunophenotyping of the BAL was additionally performed. Results included absolute cell counts and relative percentages of neutrophils, lymphocytes, macrophages/monocytes, eosinophils, basophils, plasma cells and epithelial cells.

### Outcomes

The primary endpoint of this retrospective study was whether BAL analysis including microbiological and cell analysis correlated with the detection of ACR in cryobiopsies. Therefore, we compared patients with histologically proven ACR to all patients without proven ACR. Additionally, we contrasted those with histologically proven ACR to those who clinically met the criteria of ACR but did not provide histological evidence. Secondary endpoints were to evaluate if mucosal biopsy provides ACR and correlates with histopathological results of transbronchial specimens obtained with cryoprobes and whether manual microscopic or automated BAL cell analysis is more suitable.

### Statistical analysis

Continuous outcomes were summarized using mean±SD or median (interquartile range) as appropriate. No missing values were imputed. Prespecified regression analysis included variables already used for clinical judgment. Categorical data were compared using Pearson’s χ2 or Fisher’s exact test as appropriate, and inference for stratified categorical data. For continuous data, between-group comparisons were performed using Student’s t-test for parametric variables or the Mann-Whitney U test for non-parametric variables. All statistical analyses were conducted using STATA software version 18 (StataCorp LLC). A 2-tailed P-value of 0.05 was considered statistically significant. The ethics committee of the Canton of Zurich approved the study (BASEC-ID 2020–01868 and 2021–00466). The study was consistent with the ISHLT ethical statement. All participants provided written informed consent.

## Results

A total of 240 procedures in 143 LTR (mean age 56.5 ± 9.4 years; 46.2% female, 53.8% male) at the University Hospital of Zurich were assessed. 21.2% of the bronchoscopies were performed for clinical indications. ACR was confirmed in 43 cases (17.9%) using transbronchial cryobiopsy **(**Table 1**)**. 38 LTR (15.8%) met the criteria of ACR due to lung function decline or clinical symptoms but did not show histological evidence. Patients diagnosed with ACR exhibited a significantly higher percentage of lymphocytes in their BAL compared to all LTR without histologically proven ACR (median 4% vs. 3%, p-value < 0.001) **(**Table 2, Table 3**)**. A significant correlation between lymphocyte percentage and ACR severity was observed (r=0.385, p<0.001). Using the Youden’s J optimization method, a lymphocyte threshold of 5.74% achieved an AUC of 0.715 (95% CI 0.613–0.817), with a positive predictive value of 66.7% and a negative predictive value of 76.5%. We have neither seen a relevant difference of BAL cell count nor of BAL neutrophil and eosinophil proportion between LTR with or without ACR. A low epithelial cell count together with high recovery rates confirms the high quality of our BAL collection technique.We also performed a sensitivity analysis comparing LTR who met the clinical criteria for ACR but lacked histological confirmation (n = 38) with asymptomatic patients without histologically proven ACR (n=151). This analysis revealed no associations for macrophages, neutrophils, lymphocytes, or eosinophils (neither in the manual nor in the automated cell count results).

**Table 1:** Baseline Characteristics of the Patients

**Lung Transplant Recipients, n**	**143**
Age, yr±SD	56.5±9.4
Sex - n (%)	
Female	66 (46.2)
Male	77 (53.8)
Disease - n (%)	
COPD	86 (60.1)
Sarcoidosis	7 (4.9)
Interstitial lung disease	23 (16.1)
Cystic fibrosis	9 (6.3)
Sjögren Syndrome	6 (4.2)
Bronchiolitis	7 (4.9)
Pulmonary hypertension	4 (2.8)
Pulmonary venoocclusive disease	1 (0.7)
Type of lung transplantation - n (%)	
Bilateral	129 (90.0)
Unilateral	14 (10.0)
Number of procedures - n	**240**
Days since lung transplantation, d	224 [134−380]
Side of biopsy - n (%)	
Right	130 (54.2)
Left	110 (45.8)
Air trapping on chest CT - n (%)	35 (14.6)
ACR diagnosis - n (%)	43 (17.9)
Grade of ACR - n (%) A0 / A1 / A2 / A3 / A4	197 (82.1) / 18 (7.5) / 23 (9.6) / 2 (0.8) / 0(0)
Indication bronchoscopy - n (%)	51 (21.2)
LTR who meet ACR criteria but were not histologically proven	38 (15.8%)
Hemoglobin, G/L	113 [101−122]
Leukocytes, G/L	6.5 [4.6–8.7]
Lymphocytes, G/L	0.8 [0.5–1.2]
Thrombocytes, G/L	245 [199−312]
INR	1.0 [0.9–1.0]
C-reactive protein, mg/l	0.9 [0.6–2.2]
FEV1, liters	2.3 [1.8–3.0]
DLCO, %pred.	64 [52–77]

Values are displayed as n (%) or mean±standard deviation. Values in square brackets are median [interquartile range]. Abbreviations: COPD, chronic obstructive pulmonary disease; LTR, lung transplant recipients; INR, international normalized ratio; FEV1, forced expiratory volume in the first second; DLCO, diffusing capacity for carbon monoxide. Hematological blood results originate from automated differential blood cell counts.

**Table 2:** BAL Results by ACR Diagnosis

	**No ACR**	**ACR**	**Total**	**p-value**
N (%)	197 (82.1%)	43 (17.9%)	240 (100.0%)	
BAL volume, ml	200 [150−200]	200 [150−200]	200 [150−200]	0.279
BAL recovery, ml	90 [70−105]	97 [87−115]	70 [70−110]	0.065
Manual cell results				
Cells/μl	151 [95−229]	166 [76−254]	151 [94−239]	0.439
>300 cells /μl, n (%)	67 (33.3)	11 (25.0)	77 (32.2)	0.718
Macrophages (%)	94 [89–96]	91 [83–94]	94 [89–95]	0.084
Neutrophiles (%)	3 [1–6]	3 [2–5]	3 [2–6]	0.991
Lymphocytes (%)	3 [2–4]	4 [3–11]	3 [2–5]	**<0.001**
Eosinophiles (%)	0 [0−0]	0 [0−0]	0 [0−0]	0.807
Microscopic fungal elements, n (%)	1 (1.0%)	0 (0.0%)	1 (0.9%)	0.554
Automated cell results				
Cells/μl	161 [105−246]	171 [96−287]	165 [103−248]	0.463
>300 cells /μl, n (%)	67 (33.3)	18 (41.7)	83 (34.5)	0.683
Macrophages (%)	90 [83–95]	86 [74–96]	91 [81–96]	0.450
Neutrophiles (%)	3 [1–8]	2 [1–17]	3 [1–8]	0.658
Lymphocytes (%)	3 [2–5]	3 [2–7]	3 [2–5]	0.055
Eosinophiles (%)	0 [0−0]	0 [0−0]	0 [0−0]	0.985
Epithelium cells(%)	2.5 [1.0–4.0]	3.0 [2.0–4.5]	2.5 [1.3–4.0]	0.699

**Table 3:** Multivariable Regression Analysis for ACR Diagnosis

	Coefficient	Standard Error	p-value
Cells/μl in BAL	<0.000	0.0001	0.573
% Lymphocytes in BAL	**0.022**	**0.006**	**<0.001**
Any bacterial detection in BAL	0.096	0.076	0.212
Any fungal detection in BAL	0.080	0.086	0.757
Any viral detection in BAL	0.099	0.100	0.326

Note: The manual cytological lymphocyte count for this model was chosen.

While any bacterial presence in BAL was more frequent in patients with ACR (79% vs. 64%), this association did not reach statistical significance (p-value = 0.057). However, Streptococcus parasanguinis was found significantly more often in ACR cases (p-value = 0.027), suggesting a potential association **(**Table S1**)**. Fungal or viral detections showed no significant correlation with ACR **(**Table S2 and S3**)**. Both manual and automated BAL cell counting methods yielded comparable results. However, manual lymphocyte counts were more strongly correlated with ACR and showed more accuracy and were therefore selected for the regression model (Table 2, Table 3). Mucosal biopsy did not detect ACR or reflect lymphocytic airway inflammation.

## Discussion

Elevated lymphocyte counts found in the BAL after LT are predictive of ACR, supporting their potential clinical utility in early rejection surveillance. However, microbiological, viral, or fungal analysis in BAL as well as mucosal biopsies showed no correlation with ACR found in CB. In terms of accuracy, manual BAL cell count depicted greater reliability compared to automated BAL cell count. No increase in BAL eosinophils or neutrophils was noted in this context.

A central question is whether BAL, as a less invasive procedure, can substitute for transbronchial biopsy in the diagnosis or exclusion of ACR. In BAL, following LT, elevated white blood cell counts and increased proportions of T cells, B cells, CD8⁺ cells, neutrophils, lymphocytes, eosinophils, basophils, CD25⁺ cells, and CD8⁺CD57⁺ cells were correlated with higher grade of ACR in pathological specimens.^7^ The study group of Slebos did not identify significant differences in BAL lymphocyte subtypes of LTRs with ACR compared to control subjects.^8^ During episodes of ACR, BAL samples have demonstrated altered cytokine profiles, with elevated concentrations of CXCL10, IL-1, IL-6, IL-15, and IL-17.^11^ Although neither immunophenotyping nor the detection of BAL cytokines was performed in our BAL analysis, LTRs with ACR exhibited a significantly increased percentage of lymphocytes in their BAL (median 4% vs. 3%, p-value < 0.001). Furthermore, a significant correlation was identified between BAL lymphocyte percentage and the severity of ACR (r = 0.385, p < 0.001). A lymphocyte threshold of 5.74% yielded a ROC AUC of 0.715 (95% CI: 0.613–0.817). Recent studies have explored the potential role of neutrophils in the development and progression of obliterative bronchiolitis.^9^ Furthermore, BAL eosinophil count ≥2% has been associated with an increased risk of chronic lung allograft dysfunction and decreased overall survival.^24^ One possible explanation for the absence of a correlation between neutrophil and eosinophil counts in BAL and ACR diagnosis in our analysis is that our analysis focused on ACR rather than chronic lung allograft dysfunction (CLAD) obliterative bronchiolitis syndrome (BOS), being rejection phenotypes that occur mostly beyond the first year posttransplant. Lymphocytosis in BAL appears to be more specific for ACR, whereas elevations in eosinophils and neutrophils may also occur in the context of infection.^7^ Notably, eosinophils may be absent in over 90% cases of ACR.^7^ Whether lymphocyte-predominant ACR evolves into a neutrophil- or eosinophil-predominant profile in the context of chronic rejection remains an open question and warrants further investigation. First hints for the lymphocyte-to-neutrophil shift in BAL cellular profiles originate from a prospective study involving heart and lung transplant recipients.^25^

Bacteria in the lung may elicit an inflammatory response that potentiates the risk for allograft rejection.^26^ Lung microbiota differs between patients who develop chronic lung allograft dysfunction and healthy survivors.^27^ Bacterial, viral, or fungal detection was not associated with the occurrence of ACR in our cohort (Table S1-S3). However, Streptococcus parasanguinis was found significantly more often in ACR cases (p-value = 0.027), suggesting a potential association **(**Table S1**)**. Other studies have implicated Pseudomonas aeruginosa as a potential trigger for antibody-mediated rejection.^28^ Viral infections of the respiratory tract are recognized as a contributing risk factor for the development of CLAD.^29^

A key limitation in BAL research involving LTR is the lack of standardized and detailed reporting of BAL collection methodologies. The procedure for retrieving BAL samples may differ across lung transplant centers. Our cohort adheres to the consensus statement for the standardization of BAL in LTR and is therefore suitable for inclusion in future comparative research.^15^

Currently, there is insufficient evidence to establish a standardized approach for determining BAL cell counts. According to the ISHLT consensus statement 2020, most lung transplant centers use cell count, differential, and/or microscopic analysis.^15^ We used two different cell analysis techniques in our cohort for samples from the same examination. Manual BAL cell analysis depicted greater reliability compared to automated BAL cell count (Table 2). Therefore, we recommend using manual BAL cell analysis for bronchoscopy following LT.

In addition to the BAL analysis, there is also the question of whether a mucosal biopsy can be used as a diagnostic sample indicating the presence of ACR. In 38.4% of LTR, biopsy-proven lymphocytic bronchiolitis (B-grade ACR) was observed prior to the development of CLAD, with exposure to air pollution identified as a potential predisposing factor. Notably, bronchial inflammation following exposure to airway pollutants was comparably detected in both transbronchial and bronchial mucosal biopsies.^30^ We found no correlation between findings from mucosal biopsies and ACR identified in CB. This may be attributable to a higher prevalence of endobronchial lymphocytic bronchitis in LTR with preexisting CLAD, as reported in previous studies, a population that was not included in our cohort.^20^ Currently, there is no standardized protocol for the interpretation of the samples and for obtaining endobronchial mucosal biopsies, including guidance on the optimal number of samples or their ideal anatomical location. Further molecular analyses of mucosal biopsies are underway and may help refine their diagnostic and prognostic utility in LTR.^19^

Our study has several limitations, including its retrospective design, being conducted at a single center, and the lack of longitudinal correlation with clinical outcomes and histological results, which may limit the generalizability of the findings.

Furthermore, ACR was only found in 17.9% of LTR, predominantly consisting of grade 1 and 2 ACR, which may be related to our highly standardized immunosuppression protocol.^31^ The greatest strength of our study is the use of transbronchial cryobiopsy for the detection of ACR, allowing for a more precise histopathological assessment compared to previous studies that primarily relied on conventional forceps biopsies.^2^, ^4^ Therefore, we did not report correlations of BAL with forceps biopsy. Furthermore, we analysed all detected bacterial, fungal and viral pathogens to assess potential correlation with ACR in a relatively large cohort comprising a total of 240 procedures.

For detecting ACR in LTR, transbronchial biopsy remains the gold standard. An elevated lymphocyte count above 5.74% in BAL may indicate ACR. This threshold was derived using the Youden’s J optimization method based on ROC analysis of, (mostly surveillance bronchoscopies asymptomatic LTR). Microbiological results did not correlate with ACR. Studies using BAL and mucosal biopsies, which involve immunophenotyping and cytokine profiling, need more research to confirm their usefulness in diagnosis. Importantly, even minimal acute cellular rejection (grade A1), although often clinically silent, has been independently associated with an increased risk of CLAD.^32^ In this context, emerging non-invasive biomarkers such as donor-derived cell-free DNA may provide complementary value by enabling the detection of early graft injury before functional decline or histological changes become apparent.^33^

## Ethics Statement

The study was performed in the Department of Pulmonology at the University Hospital Zurich, Zurich, Switzerland, and approved by the Cantonal Ethics Committee in Zurich, Switzerland (ID-2020–01868 and 2021–00466).

## Author Contributions

C. Steinack, A. Hillinger and T. Gaisl had full access to all data in the study and take responsibility for the integrity of the data and the accuracy of the data analysis. Study concept and design: C. Steinack, M. M. Schuurmans, T. Gaisl. Acquisition of data: C. Steinack, A. Hillinger, T. Gaisl, M. Roeder, S.M. Vesenbeckh, J.H. Rüschoff, M. Haberecker. Analysis or interpretation: C. Steinack, H. Rüschoff, R. Hage, D. Lenggenhager, S. Ulrich, M. Haberecker, T. Gaisl. Drafting of the manuscript: C. Steinack, A. Hillinger, T. Gaisl. Critical revision of the manuscript for important intellectual content: All authors. Statistical analysis: T. Gaisl. Administrative, technical, or material support: C. Steinack, J.H. Rüschoff, M. Haberecker, M. Kohler T. Gaisl. Study supervision: M.M. Schuurmans.

## Financial Disclosure Statement

This study has no funding.

## Declaration of Interest Statement

The authors declare the following financial interests/personal relationships which may be considered as potential competing interests: M. Kohler received consulting fees from Novartis. He is co-founder and shareholder of Deep Breath Intelligence AG, a company that provides services in the field of breath analysis

## Declaration of Generative AI and AI-assisted technologies in the writing process

During the preparation of this work the authors used *ChatGPT* to improve readability. After using this tool, the authors reviewed and edited the content as needed and take full responsibility for the content of the published article.

## Footnotes

Appendix A Supplementary data associated with this article can be found in the online version at doi:10.1016/j.jhlto.2026.100582.

## References

[1] Chambers D.C., Cherikh W.S., Harhay M.O. (2019). The International Thoracic Organ Transplant Registry of the International Society for Heart and Lung Transplantation: Thirty-sixth adult lung and heart-lung transplantation Report-2019; Focus theme: Donor and recipient size match. J Heart Lung Transplant.

[2] Steinack C., Gaspert A., Gautschi F. (2022). Transbronchial cryobiopsy compared to forceps biopsy for diagnosis of acute cellular rejection in lung transplants: analysis of 63 consecutive procedures. Life (Basel).

[3] Mohamed S., Mendogni P., Tosi D. (2020). Transbronchial cryobiopsies in lung allograft recipients for surveillance purposes: initial results. Transplant Proc.

[4] Steinack C., Roeder M., Vesenbeckh S. (2025). Transbronchial cryobiopsy alone versus combined with traditional forceps biopsy for acute cellular rejection in lung transplant recipients. A diagnostic randomized trial. JHLT Open.

[5] Roden A.C., Kern R.M., Aubry M.C. (2016). Transbronchial cryobiopsies in the evaluation of lung allografts: do the benefits outweigh the risks?. Arch Pathol Lab Med.

[6] Gershman E., Ridman E., Fridel L. (2018). Efficacy and safety of trans-bronchial cryo in comparison with forceps biopsy in lung allograft recipients: analysis of 402 procedures. Clin Transplant.

[7] Greenland J.R., Jewell N.P., Gottschall M. (2014). Bronchoalveolar lavage cell immunophenotyping facilitates diagnosis of lung allograft rejection. Am J Transplant.

[8] Slebos D.J., Postma D.S., Koeter G.H., Van Der Bij W., Boezen M., Kauffman H.F. (2004). Bronchoalveolar lavage fluid characteristics in acute and chronic lung transplant rejection. J Heart Lung Transplant.

[9] Reynaud-Gaubert M., Thomas P., Badier M., Cau P., Giudicelli R., Fuentes P. (2000). Early detection of airway involvement in obliterative bronchiolitis after lung transplantation. Functional and bronchoalveolar lavage cell findings. Am J Respir Crit Care Med.

[10] Speck N.E., Schuurmans M.M., Murer C., Benden C., Huber L.C. (2016). Diagnostic value of plasma and bronchoalveolar lavage samples in acute lung allograft rejection: differential cytology. Respir Res.

[11] Speck N.E., Schuurmans M.M., Benden C., Robinson C.A., Huber L.C. (2017). Plasma and bronchoalveolar lavage samples in acute lung allograft rejection: the potential role of cytokines as diagnostic markers. Respir Res.

[12] Speck N.E., Probst-Muller E., Haile S.R. (2020). Bronchoalveolar lavage cytokines are of minor value to diagnose complications following lung transplantation. Cytokine.

[13] Weigt S.S., Wang X., Palchevskiy V. (2019). Usefulness of gene expression profiling of bronchoalveolar lavage cells in acute lung allograft rejection. J Heart Lung Transplant.

[14] Koutsokera A. (2019). Rethinking bronchoalveolar lavage in acute cellular rejection: How golden is the standard of transbronchial biopsies?. J Heart Lung Transplant.

[15] Martinu T., Koutsokera A., Benden C. (2020). International Society for Heart and Lung Transplantation consensus statement for the standardization of bronchoalveolar lavage in lung transplantation. J Heart Lung Transplant.

[16] Bratke K., Weise M., Stoll P., Virchow J.C., Lommatzsch M. (2024). Flow cytometry as an alternative to microscopy for the differentiation of BAL fluid leukocytes. Chest.

[17] Meyer K.C., Raghu G., Baughman R.P. (2012). An official American Thoracic Society clinical practice guideline: the clinical utility of bronchoalveolar lavage cellular analysis in interstitial lung disease. Am J Respir Crit Care Med.

[18] Du Rand I.A., Blaikley J., Booton R. (2013). British Thoracic Society guideline for diagnostic flexible bronchoscopy in adults: accredited by NICE. Thorax.

[19] Halloran K., Vos R., Snell G., Greenland J.R. (2025). The lung transplant endobronchial biopsy: A forgotten specimen comes of age. J Heart Lung Transplant.

[20] Greenland J.R., Jones K.D., Hays S.R. (2013). Association of large-airway lymphocytic bronchitis with bronchiolitis obliterans syndrome. Am J Respir Crit Care Med.

[21] Schumann C., Hetzel J., Babiak A.J. (2010). Cryoprobe biopsy increases the diagnostic yield in endobronchial tumor lesions. J Thorac Cardiovasc Surg.

[22] Babiak A., Hetzel J., Krishna G. (2009). Transbronchial cryobiopsy: a new tool for lung biopsies. Respiration.

[23] Gershman E., Fruchter O., Benjamin F. (2015). Safety of cryo-transbronchial biopsy in diffuse lung diseases: analysis of three hundred cases. Respiration.

[24] Verleden S.E., Ruttens D., Vandermeulen E. (2014). Elevated bronchoalveolar lavage eosinophilia correlates with poor outcome after lung transplantation. Transplantation.

[25] Tikkanen J., Lemstrom K., Halme M., Pakkala S., Taskinen E., Koskinen P. (2001). Cytological monitoring of peripheral blood, bronchoalveolar lavage fluid, and transbronchial biopsy specimens during acute rejection and cytomegalovirus infection in lung and heart--lung allograft recipients. Clin Transplant.

[26] Tanaka S., Gauthier J.M., Terada Y. (2021). Bacterial products in donor airways prevent the induction of lung transplant tolerance. Am J Transplant.

[27] Combs M.P., Wheeler D.S., Luth J.E. (2021). Lung microbiota predict chronic rejection in healthy lung transplant recipients: a prospective cohort study. Lancet Respir Med.

[28] Liao F., Zhou D., Cano M. (2025). Pseudomonas aeruginosa infection induces intragraft lymphocytotoxicity that triggers lung transplant antibody-mediated rejection. Sci Transl Med.

[29] Gunasekaran M., Bansal S., Ravichandran R. (2020). Respiratory viral infection in lung transplantation induces exosomes that trigger chronic rejection. J Heart Lung Transplant.

[30] Verleden S.E., Scheers H., Nawrot T.S. (2012). Lymphocytic bronchiolitis after lung transplantation is associated with daily changes in air pollution. Am J Transplant.

[31] Schuurmans M.M., Raeber M.E., Roeder M., Hage R. (2023). Adaptive immunosuppression in lung transplant recipients applying complementary biomarkers: the Zurich protocol. Medicina (Kaunas).

[32] Shino M.Y., Weigt S.S., Li N. (2017). Impact of allograft injury time of onset on the development of chronic lung allograft dysfunction after lung transplantation. Am J Transplant.

[33] Rosenheck J.P., Ross D.J., Botros M. (2022). Clinical validation of a plasma donor-derived cell-free DNA assay to detect allograft rejection and injury in lung transplant. Transplant Direct.

